# GABAergic Interneurons in Seizures: Investigating Causality With Optogenetics

**DOI:** 10.1177/1073858418805002

**Published:** 2018-10-15

**Authors:** Vincent Magloire, Marion S. Mercier, Dimitri M. Kullmann, Ivan Pavlov

**Affiliations:** 1 Department of Clinical and Experimental Epilepsy, Institute of Neurology, UCL, London, UK

**Keywords:** epilepsy, seizures, interictal spikes, interneurons, optogenetics

## Abstract

Seizures are complex pathological network events characterized by excessive and hypersynchronized activity of neurons, including a highly diverse population of GABAergic interneurons. Although the primary function of inhibitory interneurons under normal conditions is to restrain excitation in the brain, this system appears to fail intermittently, allowing runaway excitation. Recent developments in optogenetics, combined with genetic tools and advanced electrophysiological and imaging techniques, allow us for the first time to assess the causal roles of identified cell-types in network dynamics. While these methods have greatly increased our understanding of cortical microcircuits in epilepsy, the roles played by individual GABAergic cell-types in controlling ictogenesis remain incompletely resolved. Indeed, the ability of interneurons to suppress epileptic discharges varies across different subtypes, and an accumulating body of evidence paradoxically implicates some interneuron subtypes in the initiation and maintenance of epileptiform activity. Here, we bring together findings from this growing field and discuss what can be inferred regarding the causal role of different GABAergic cell-types in seizures.

## Background

Epilepsy affects approximately 1% of the population, and in developed countries up to 30% of patients continue to experience seizures despite optimal antiepileptic medication (Schmidt and Loscher 2005). There is therefore an urgent need to identify novel therapeutic targets and develop new treatment strategies. Focal seizures are widely considered to arise from a disturbance of the excitation/inhibition balance, and in particular a failure of the GABAergic inhibitory system. In support of this view, reducing inhibition experimentally by blocking GABAergic neurotransmission induces epileptiform activity both in vitro and in animal models (Pitkänen and others 2005), while drugs that potentiate inhibition suppress seizures and are widely used clinically (Mula 2011). Furthermore, a breakdown of feed-forward inhibition has been shown to occur during the propagation of the seizure front across the cortex (Schevon and others 2012; Trevelyan and others 2006; Trevelyan and others 2007). Although the evidence for a failure of GABAergic inhibition is compelling, it must be interpreted in the context of a highly diverse population of interneurons. Indeed, more than 20 different interneuron subtypes have been identified in the cortex, displaying a wide range of electrophysiological properties, morphologies, genetic markers, innervation patterns and GABAergic signaling profiles (Ascoli and others 2008; Jiang and others 2015). Molecular markers, such as the calcium-binding protein parvalbumin (PV) and the neuropeptides somatostatin (SOM) and vasointestinal peptide (VIP), have been used to distinguish between interneuron subtypes that primarily mediate somatic inhibition, dendritic inhibition, and disinhibition, respectively (Box 1, Tremblay and others 2016). Advances in Cre-Lox technology, optogenetics, and imaging methods allow these different types of inhibition to be selectively manipulated, enabling a cellular and spatiotemporal resolution that was not previously achievable using pharmacological agents. However, while optogenetic inhibition of principal neuron activity has been successfully employed to curtail seizures in different models (Chiang and others 2014; Krook-Magnuson and others 2013; Paz and others 2012; Wykes and others 2012), optogenetic manipulation of interneuronal activity has thus far generated mixed results, which may in part be due to the dynamic nature of epileptic states. In this review, we aim to bring together these apparently conflicting findings, and to explain them in the light of key experimental differences: specifically, we distinguish between studies investigating interictal discharges and those that address seizures per se, and between findings on the generation of seizure activity and on its maintenance. We also compare the involvement of different GABAergic cell subtypes and examine the various optogenetic stimulation protocols that have been used.

Box 1. Cortical inhibition. Different types of cortical interneurons, identified based on the expression of specific molecular markers, have distinct postsynaptic targets.

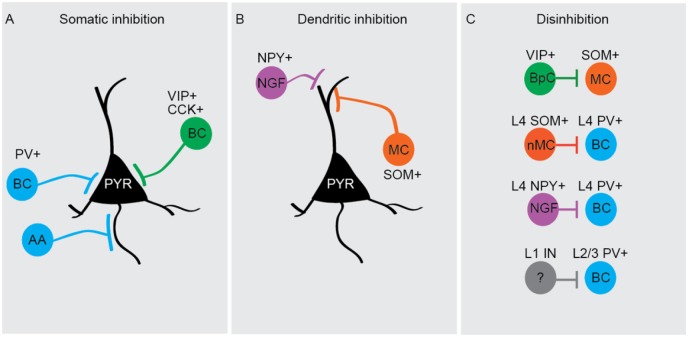

(A) Somatic inhibition is primarily mediated by PV+ (parvalbumin-positive) interneurons (blue), which represent nearly 40% of all neocortical interneurons and consist mostly of fast-spiking basket cells (BCs; Tremblay and others 2016). They form numerous synapses onto the perisomatic region of pyramidal neurons and exhibit unusually high spiking frequencies. The unique firing properties of these cells, and their widespread synaptic contacts, located close to the action potential initiation site, enables PV+ BCs to exert powerful inhibitory control over the output of pyramidal neurons (Hu and others 2014). PV+ interneurons also comprise axo-axonic (AA) or “chandelier” cells, which form synapses with the axon initial segment of pyramidal cells (Taniguchi and others 2012). An additional type of BC expressing cholecystokinin (CCK+) and vasointestinal peptide-expressing (VIP+; green) also mediates somatic inhibition and features regular or burst-firing properties (Tremblay and others 2016).

(B) SOM+ (somatostatin) interneurons (30% of all interneurons, orange), consisting primarily of Martinotti cells (MC) in the cortex, represent the major source of dendritic inhibition (Tremblay and others 2016). These cells form synapses not only onto the dendrites of pyramidal neurons but also onto those of other inhibitory cell-types. While the net contribution of SOM+ interneurons to pyramidal neuron inhibition is somewhat smaller than that of PV+ interneurons (Pfeffer and others 2013), their efficient recruitment by local pyramidal neurons leads to feedback inhibition that is powerful enough to suppress dendritic Ca^2+^ spikes, and the consequent generation of action potential bursts, in neighboring principal neurons (Murayama and others 2009; Silberberg and Markram 2007). An additional source of dendritic inhibition comes from neuropeptide Y-expressing (NPY^+^) neurogliaform (NGF) neurons. They have a high connection probability spanning across cell types and cortical layers (Jiang and others 2015), and form unconventional synapses generating a unique form of GABAergic transmission known as “volume transmission” (Oláh and others 2009). SOM+ MCs and NPY^+^ NGF neuron inhibition involve GABA_A_ and GABA_B_ signaling (Tremblay and others 2016), and by connecting to all other neurons and across cortical layers, have been described as “master regulators” of cortical microcircuit excitability (Jiang and others 2015).

(C) Disinhibition occurs when the net inhibitory effect of a certain type of GABAergic cell is greater on interneurons than it is on principal cells. The most well-characterized “disinhibitory” cells are bipolar cells (BpC) expressing VIP, so called VIP+ neurons, which primarily inhibit SOM+ interneurons. Three other disinhibitory circuits, each targeting PV+ interneurons, have been identified so far: non-Martinotti (nMC) SOM+ cells in layer 4 of the barrel cortex specifically and strongly inhibit PV+ interneurons from the same layer (Tremblay and others 2016), NGF cells in layer 4 suppress the feed-forward inhibitory action of PV+ interneurons onto layer 4 stellate cells (Chittajallu and others 2013), and interneurons from layer 1 (L1 IN) of the neocortex disinhibit the cortical circuit by suppressing the inhibitory activity of layer 2/3 PV+ interneurons (Letzkus and others 2011).

## Epilepsy and Interneurons

The extensive literature on epilepsy and interneurons covers multiple phenomena occurring in epileptic patients, from the mechanisms underlying epileptogenesis and associated structural changes, to the role of high frequency oscillations in the maintenance of ictal discharges. Here, we focus on studies that have employed optogenetic and imaging techniques to target and manipulate GABAergic interneurons and review what these techniques have revealed about the causal role of these cells in interictal discharges, and in the generation and maintenance of seizures.

### Interneurons: Sufficient for Interictal Discharges

**Figure 1.**
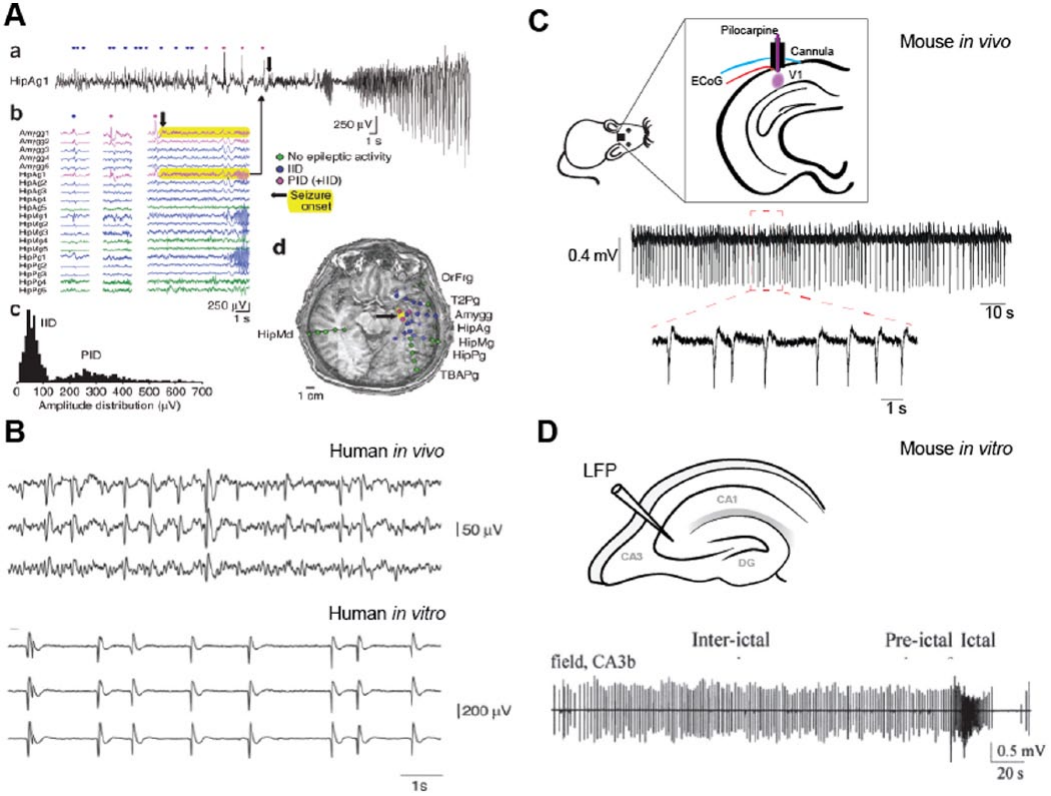
Interictal spikes. (A) Three main types of activity observed in a human electroencephalography (EEG) recording. (a, b) Intracranial EEG traces showing interictal discharges (IID, blue), pre-ictal discharges (PID, pink) and ictal activity (yellow). (c) Amplitude distribution of IID and PID. (d) Spatial localization of each recording electrode and associated activity, showing a core of ictal activity (yellow spots) surrounded by IIDs (blue spots). Modified from Huberfeld and others (2011). (B) In vivo and in vitro recordings of interictal spikes in human mesial temporal lobe. Modified from Cohen and others (2002). (C) In vivo electrocorticogram (ECoG) recording of interictal spikes in a pilocarpine neocortical focal epilepsy mouse model (unpublished data). (D) In vitro local field potential (LFP) recording of interictal spikes in hippocampal slices superfused with high K^+^ solutions. Modified from Dzhala and Staley (2003).

Epileptic cortical microcircuits commonly exhibit fast (tens of milliseconds), high-amplitude electrographic signals in between seizures (Fig. 1). These intermittent discharges, which have few or no clinical manifestations, are often used clinically to support a diagnosis of epilepsy (de Curtis and Avanzini 2001). Work on rodent and human cortical slices superfused with chemoconvulsant solutions (e.g., 4-aminopyridine [4-AP] and/or low Mg^2+^/high K^+^) has revealed two different types of interictal activity (Avoli and de Curtis 2011; Cohen 2002; Dzhala and Staley 2003; Huberfeld and others 2011), also seen in epileptic patients (Huberfeld and others 2011). Typical “interictal spikes” rely on both glutamatergic and GABAergic transmission and can be recorded at sites distant from the focus; the second type of activity, termed “pre-ictal spikes,” is primarily glutamatergic, spatially restricted to the seizure focus, and precedes seizures, hence its name (Avoli and de Curtis 2011; Huberfeld and others 2011; Trevelyan and others 2006; Zhang and others 2012). Since GABAergic transmission plays a lesser role in the generation of pre-ictal spikes, we will not discuss these network events further, and will focus instead on interictal spikes.

Evidence for the involvement of the GABAergic system in generating interictal spikes comes from several sources. First, blocking ionotropic glutamate receptors alone does not suppress interictal spikes, while a combination of glutamate and GABA_A_ receptor antagonists, in in vitro models, does (Avoli and de Curtis 2011; Bohannon and Hablitz 2018; Chang and others 2018; Huberfeld and others 2011; Trevelyan and others 2006). Second, analysis of the temporal relationship between neuronal activity and interictal episodes in humans revealed that activity in putative interneurons precedes interictal discharges, while pyramidal neuron activity coincides with their onset (Huberfeld and others 2011). Similar observations were recently made using *in vivo* two-photon imaging of the genetically encoded calcium indicators GCaMP5 or GCaMP6 in a pilocarpine mouse model of temporal lobe epilepsy (TLE; Muldoon and others 2015). Imaging single cell transient calcium signals as a proxy for spiking activity in the hippocampus, the authors noticed that the majority of calcium signals observed during interictal spikes emanated from the *stratum oriens*, a layer mainly containing interneurons. In contrast, only a small percentage of neurons in the *stratum pyramidale*, consisting mostly of pyramidal cells, were active (Muldoon and others 2015). An insight into the temporal order in which neurons fire during interictal discharges comes from a study by Karlócai and others (2014), who recorded from different classes of interneurons in hippocampal slices perfused with high K^+^, 4-AP, low Mg^2+^, or the GABA_A_ receptor antagonist gabazine. While pyramidal neurons fired only at the peak of the spontaneous burst discharges, fast-spiking PV-expressing (PV+) basket cells (BCs) fired mainly at the onset. In contrast, most of the recorded axo-axonic (AA) cells, which also express PV, increased their firing throughout the interictal discharges, as did cholecystokinin-expressing (CCK+) BCs and dendrite-targeting interneurons (Karlócai and others 2014).

Some of the strongest evidence implicating interneurons in the generation of interictal activity comes from direct activation of GABAergic neurons using optogenetic tools. Thus, using the excitatory opsin channelrhodopsin2 (ChR2), expressed in the pan-GABAergic Cre driver mouse line Gad2-Cre, Ledri and others (2014) found that light pulses delivered in the presence of 4-AP and low Mg^2+^ in vitro could induce burst-firing of CA3 pyramidal cells. While subsequent bursts were delayed, it is unclear whether this was due to a refractory period imposed by the initial light-evoked discharge, or to a biphasic effect of stimulating the interneurons, initially recruiting principal cells and then inhibiting them (Ledri and others 2014). The ability to trigger burst-discharges by photo-depolarizing interneurons was confirmed by Yekhlef and others (2015) working on entorhinal cortical slices superfused with 4-AP, who further showed that optogenetic stimulation of either PV+ or SOM+ interneurons was equally effective. In neocortical slices, Chang and others (2018) were also able to evoke interictal activity using another mouse line expressing ChR2 in interneurons, Vgat-ChR2, and, importantly, found that while blocking excitatory transmission partially prevented the induction of this activity, only GABA_A_ receptor antagonists abolished it completely (Chang and others 2018). Complementing this finding, Bohannon and Hablitz (2018) reported that hypersynchronous GABAergic activity could be triggered in the absence of fast glutamatergic excitation; this occurred whether optogenetic stimulation was restricted to PV+ or SOM+ interneurons, although photo-depolarization of VIP+ interneurons was ineffective.

The optogenetic results summarized above argue that synchronous interneuron activity is sufficient to trigger burst activity, but is it necessary? Bohannon and Hablitz (2018) expressed the inhibitory opsin archaerhodopsin (Arch) in different subsets of interneurons and found that PV+ hyperpolarization strongly suppressed the generation of epileptiform bursts, while inhibition of SOM+ interneurons was only minimally effective. These results imply that while both PV+ and SOM+ interneurons are sufficient for the generation of interictal activity, only PV+ cells are necessary for its induction. However, these experiments were carried out in the presence of glutamatergic blockers, and it will therefore be important to determine whether this holds true when glutamatergic transmission is intact.

The exact mechanism by which optogenetic activation of GABAergic cells can generate interictal activity remains to be determined. For instance, simultaneous optogenetic activation of many interneurons could entrain firing of glutamatergic neurons by triggering post-inhibitory rebound excitation (Chang and others 2018; Sessolo and others 2015). Indeed, generating synchrony and maintaining network oscillations in the brain is thought to be one of the principal functions of GABAergic cells, and of PV+ interneurons in particular (Cardin and others 2009; Cobb and others 1995; Sohal and others 2009). Importantly, it has been argued that interictal activity may prevent seizure generation (Avoli and others 2002), reflecting the presence of an “inhibitory restraint” (Box 2; Trevelyan and others 2006; Trevelyan and others 2007; Trevelyan and Schevon 2012), the breakdown of which enables the transition to ictal discharges in the cortex (Cammarota and others 2013; Karlócai and others 2014; Zhang and others 2012). While this theory is attractive, it remains unclear whether interictal spikes actually serve a protective function (see Seizure Initiation; de Curtis and Avoli 2016; Staley and others 2011).

Box 2. Inhibitory restraint. Focal seizures are characterized by localized ictal discharges (red) emanating from an excitatory core, contained by a surrounding inhibitory restraint. Pathological activity in this ‘halo’ may only manifest as interictal spikes (blue; left). When inhibitory restraint fails, it allows the excitatory core to propagate (middle), which eventually may lead to seizure generalization (right).

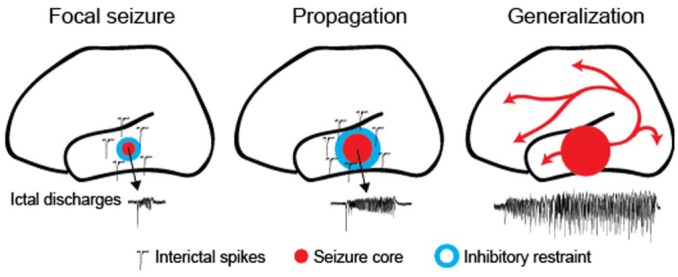

The idea of an inhibitory restraint preventing the spread of seizures has arisen from observations that seizures travel much faster when GABA_A_ receptors are blocked (Trevelyan and others 2007; Trevelyan and Schevon 2012), and that large inhibitory currents often precede seizures at the site of recorInhibitory restraint. Focal seizures are characterized by localding (Trevelyan and others 2006). Further experimental support comes from recordings in people with focal epilepsy, where an “ictal wavefront,” characterized by large-amplitude EEG signals, is surrounded by an inhibited area featuring low firing rates, termed the “ictal penumbra” (Schevon and others 2012; Smith and others 2016). Thus, massive excitation created by the ictal wavefront appears to generate strong feed-forward inhibition in areas yet to be invaded.

### Interneuron Roles in Seizure Generation and Seizure Maintenance

The cellular and network mechanisms of electrographic seizures have been the subject of intense investigation using numerous models, from acute in vitro preparations to chronic in vivo experiments. A huge body of work clearly implicates the GABAergic system in ictogenesis, and the ability to manipulate the activity of interneurons optogenetically provides us with a unique opportunity to disentangle the role of different GABAergic cells in the generation of this pathological activity. To make sense of the complex, often seemingly contradictory results in this field, we make an important distinction between those studies manipulating interneurons *before* (or in between) seizure episodes, thus exploring the mechanisms of seizure initiation, and those manipulating interneurons *during* seizures, and thus investigating their involvement in seizure maintenance.

#### Seizure Initiation

Optogenetic depolarization of interneurons has been reported to initiate cortical seizures both in vitro and in vivo in the presence of 4-AP (Assaf and Schiller 2016; Chang and others 2018; Sessolo and others 2015; Shiri and others 2015; Yekhlef and others 2015). Furthermore, stimulation of either PV+ or SOM+ cells can trigger ictal discharges in vitro (Sessolo and others 2015; Shiri and others 2015; Yekhlef and others 2015), although only PV+ interneuron stimulation has thus far been shown to be ictogenic in vivo (Assaf and Schiller 2016). The outcomes of these studies suggest that GABA-ergic neurons may have an active role in seizure generation, challenging the traditional view that seizures occur when an inhibitory restraint fails to contain runaway excitation (Box 2). Two studies have, however, reported anti-ictogenic actions of somatic- and dendritic-targeting interneurons, this time in generalized seizure models. Here, photo-activation of PV+ or SOM+ neurons was found to increase seizure threshold (Wang and others 2017), while photo-inhibition of these cells appeared to have the opposite effect (Khoshkhoo and others 2016). In addition, VIP+ cell hyperpolarization, presumably causing disinhibition of other interneuron subtypes, significantly reduced the probability of triggering a generalized seizure (Khoshkhoo and others 2016). It is worth noting, however, that both studies used electrical or optogenetic stimulation to precipitate seizures, which could differ significantly from the ictal discharges induced by chemoconvulsants such as 4-AP.

**Figure 2.**
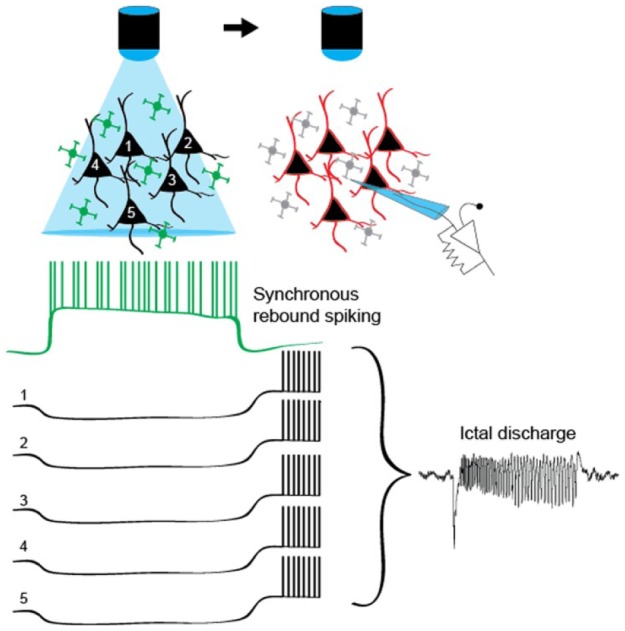
Possible mechanism of seizure induction by photo-activation of interneurons: post-inhibitory rebound spikes. Optogenetic activation of many interneurons (green) hyperpolarizes a large population of pyramidal neurons (black). When the photo-stimulation ends, pyramidal neurons are simultaneously released from inhibition and fire rebound action potentials initiating an ictal discharge.

What mechanism(s) might underlie the pro-epileptic effect of photo-stimulating PV+ and SOM+ GABAergic neurons within the epileptic network? One hypothesis, for which evidence is accumulating, is that seizures arise from post-inhibitory rebound synchronization of pyramidal neurons (Fig. 2). Photo-depolarization of interneurons, and of PV+ cells in particular, has been shown to promote synchronous firing of pyramidal neurons in vitro (Chang and others 2018; Sessolo and others 2015) and, importantly, to induce post-inhibitory rebound spikes; indeed, these were observed in up to 30% of putative pyramidal neurons recorded in vivo following the end of PV+ interneuron photo-stimulation (Assaf and Schiller 2016). Whether SOM+ interneuron recruitment can also promote such synchronous activity remains to be determined.

**Figure 3.**
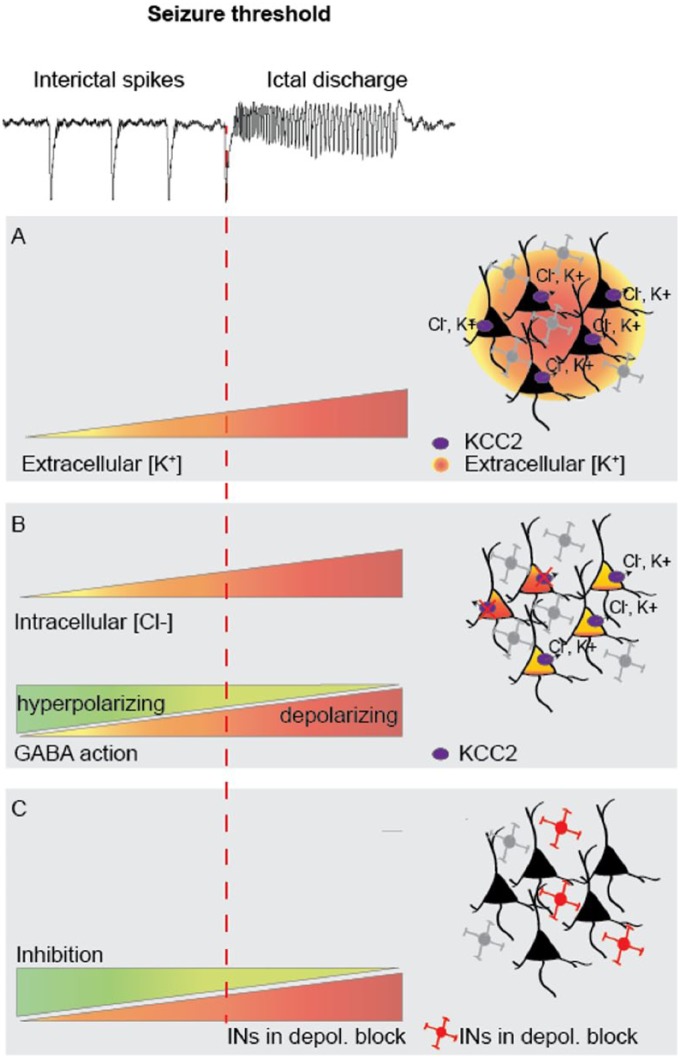
Possible mechanisms of seizure facilitation by interneurons. (A) Intense activity of interneurons during interictal bursts leads to GABA_A_ receptor–mediated Cl^−^ flux into principal neurons. This leads to KCC2-mediated efflux of K^+^ and Cl^−^. A seizure could then be triggered when extracellular K^+^ accumulation depolarizes a sufficient number of excitatory neurons. (B) When the capacity of principal neurons to extrude Cl^−^ is overwhelmed, Cl^−^ accumulation gradually shifts E_GABA_ to more depolarized potentials, weakening or even reversing the effect of GABA, and precipitating seizures. (C) Excessive activation of interneurons during interictal discharges causes them to enter a state of depolarization block. A seizure is generated when a sufficient number of interneurons cease firing and thus fail to contain excitatory activity.

Three additional possible mechanisms, which could underlie an interneuron-evoked transition from interictal to ictal discharges, have been proposed. The first is that intense GABAergic activity during interictal discharges leads to a build-up of extracellular K^+^, the concentration of which becomes sufficiently high to trigger seizures. K^+^ extrusion could occur via voltage-gated K^+^ channels recruited during intense spiking activity and/or via the neuron-specific K^+^-Cl^−^ co-transporter KCC2, following GABA_A_ receptor-mediated Cl^−^ influx into pyramidal cells (Fig. 3A) (Viitanen and others 2010). In support of this hypothesis, in vitro slice studies have shown that GABA_A_ receptor activation can induce elevations in extracellular K^+^ (Barolet and Morris 1991) and that GABA-mediated interictal activity induced by 4-AP is associated with an elevation in extracellular K^+^ (Avoli and others 1996; Librizzi and others 2017). Photo-stimulation of a large population of interneurons could therefore trigger seizures by elevating the extracellular K^+^ concentrations, and thereby depolarizing principal cells, until the seizure threshold is reached.

A second mechanism to explain how interneuron activity could trigger seizures relates to the consequences of intense Cl^−^ influx itself: elevations of intracellular Cl^−^ in pyramidal cells results in a shift in E_GABA_, which weakens the ability of GABA_A_ receptors to hyperpolarize principal neurons, or may even convert GABAergic hyperpolarization to depolarization (Fig. 3B; Cohen and others 2002; Pavlov and others 2013; Viitanen and others 2010). In support of this model, an increase in intracellular Cl^−^ was observed in pyramidal neurons during pre-ictal bursts in juvenile animals (Lillis and others 2012), and Alfonsa and others (2015) recently showed that loading pyramidal neurons with Cl^−^ using the optogenetic actuator halorhodopsin (eNpHR) can facilitate seizure generation in the presence of 4-AP. Whether interneuron-mediated Cl^−^ loading can and does induce seizures, however, remains to be shown. Indeed, GABA_A_ receptors inhibit neurons not only by hyperpolarizing them, but also by shunting excitatory currents, and so a collapse of the Cl^−^ gradient does not necessarily equate to failure of inhibition. Of course, accumulation of extracellular K^+^ and intracellular Cl^−^ are not mutually exclusive and could work in synergy to depolarize pyramidal neurons.

Finally, since GABAergic activity dominates interictal events, seizure onset is often heralded by presynaptic exhaustion of GABA release. This could arise from depletion of GABA vesicles, or excessive depolarization of interneurons, resulting in a failure to generate action potentials (depolarization block). Such a phenomenon has been proposed to occur in PV+ BCs (Cammarota and others 2013; Karlócai and others 2014; Trevelyan and others 2006; Zhang and others 2012). It is therefore possible that the pro-seizure effects of interneuron photo-stimulation may be due to the exacerbation of a breakdown of GABA-mediated inhibition, rather than to a paradoxical GABA-mediated excitation (Fig. 3C). In this scenario, photo-depolarization of a large population of interneurons would simultaneously precipitate them into depolarization block, thereby leading to a failure of inhibition and triggering a seizure.

Although some existing evidence supports the post-inhibitory rebound excitation hypothesis, no study has yet shown a causal link between interneuron activation and any of the mechanisms discussed above. Furthermore, it is important to note that all the aforementioned optogenetic studies were carried out using application of 4-AP, either alone or in combination with 0 Mg^2+^ or *N*-methyl-d-aspartate (NMDA) (Assaf and Schiller 2016; Chang and others 2018; Sessolo and others 2015; Shiri and others 2015; Yekhlef and others 2015), with only one example of interneuron-induced ictal discharges in a 0 Mg^2+^ solution alone (Chang and others 2018). This is important, as 4-AP blocks Kv3 channels (Rudy and McBain 2001), which are mainly found on interneurons, and particularly in PV+ cell dendrites and axons (Hu and others 2014). Since these channels are critical for repolarization following action potentials (Rudy and McBain 2001), blocking them may predispose PV+ interneurons to depolarization block during optogenetic stimulation. It will be important to determine whether the pro-ictogenic effects of optogenetic stimulation of interneurons occur in other models, such as brain slices exposed to high K^+^ (Karlócai and others 2014), or in animals with intracortical pilocarpine injections (Kätzel and others 2014). Ultimately, these mechanisms will need to be investigated in models of epilepsy per se, as opposed to acute models of seizures resulting from disinhibition or chemoconvulsants.

#### Seizure Maintenance

Once a seizure is established, excitation dominates and underlies propagation of the pathological activity. Interneurons are, however, likely not passive bystanders, and each of the seizure initiation mechanisms described above could also contribute to seizure maintenance. To reconcile some of the disparate findings from optogenetic studies on interneurons in seizures, three experimental variables should be taken into account. These are (1) the *frequency* of optogenetic stimulation, affecting network synchronization; (2) the *location and the timing* of optogenetic intervention (inside or outside of the focus), affecting seizure propagation; and (3) the *GABAergic cell subtype* targeted.

##### Stimulation frequency and network synchronization

**Figure 4.**
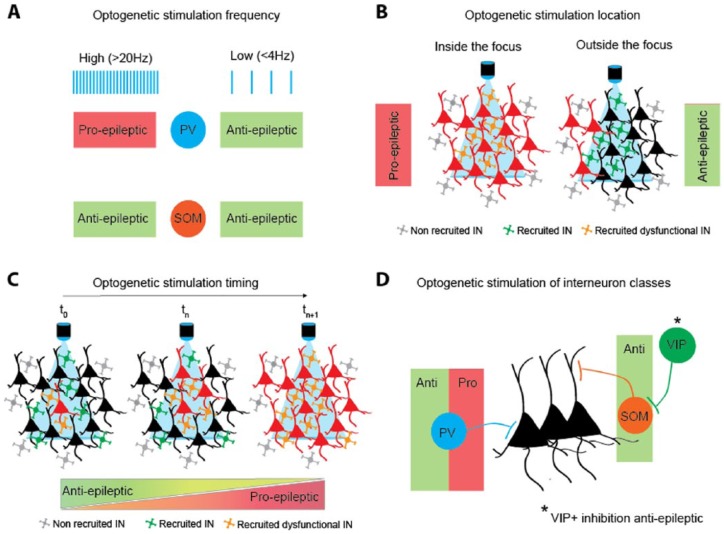
Factors influencing the effect of optogenetic stimulation of interneurons during seizures. (A) Stimulation frequency: the effect of PV+ (parvalbumin) but not SOM+ (somatostatin) cell photo-stimulation is frequency-dependent. (B) Location of stimulation relative to the seizure focus: photo-activation of interneurons within the focus is pro-ictogenic, while outside is anti-ictogenic. (C) Timing of stimulation relative to the seizure onset: optogenetic intervention before or early in the seizure can potentiate interneuron-mediated inhibition, while photo-activation at later time points—when the seizure has spread—may lead to pro-epileptic effects. (D) Interneuronal subtype: the effect of activating PV+ interneurons changes over time, while SOM+ neuron stimulation is anti-epileptic throughout seizures.

Both high- and low-frequency electrical stimulation, capable of disrupting neuronal synchrony characteristic of pathological network activity, have been shown to effectively curtail seizures in humans, as well as in various animal models (Chiang and others 2014; Jobst and others 2016; Koubeissi and others 2013). This strongly suggests that synchronization is a critical factor in seizure maintenance. In line with this, high- and low-frequency optogenetic stimulation of neurons (both excitatory and inhibitory) using Thy1-ChR2 mice, suppresses ictal discharges in both in vitro and in vivo models (Chiang and others 2014; Ladas and others 2015). Interestingly, results with selective activation of GABAergic interneurons are more complex. Thus, using Vgat-ChR2 mice, low-frequency (1 Hz) stimulation of interneurons in a focal 4-AP in vivo model was found to curtail seizures (Ladas and others 2015), but high-frequency stimulation (20 Hz) of these cells in a hippocampal kindling model was instead found to be pro-epileptic, dramatically increasing after-discharge and seizure duration (Wang and others 2017). Interneuron stimulation thus appears to either suppress or promote seizure activity in a frequency-dependent manner. This pattern, however, may not hold true for both somatic and dendritic inhibition. Indeed, whilst the anti-seizure effects of low-frequency interneuron stimulation were successfully reproduced by low-frequency stimulation of either PV+ or SOM+ neurons alone (Shiri and others 2017), the pro-seizure effects of high-frequency stimulation were only seen with PV+ cell activation (Wang and others 2017); high-frequency stimulation of SOM+ interneurons remained anti-seizure, shortening after-discharge and seizure duration (Wang and others 2017). These studies imply that somatic inhibition exerts either pro- or anti-seizure effects depending on the frequency at which it is recruited, while the effects of dendritic inhibition are anti-ictal regardless of stimulation frequency (Fig. 4A). It is important to note, however, that the above studies used different in vitro and in vivo seizure models. Replicating them in a single model, preferably in vivo, will be important to test how this principle can be generalized, and to further refine the optimal stimulation pattern to curtail seizures.

What mechanisms could underlie these frequency-dependent effects of optogenetic stimulation? Ladas and others (2015) suggest that low-frequency stimulation generates GABA-mediated bursts (resembling interictal events), which could entrain the network and thus lead to seizure termination. Indeed, highly synchronized low-frequency high-amplitude bursts, such as occur during the clonic phase of a seizure, are proposed to aid in terminating ictal discharges by creating a long refractory period which prevents further reactivation of the network (de Curtis and Avoli 2015). In line with this hypothesis, selective low-frequency photo-stimulation of PV+ or SOM+ neurons has been shown to generate synchronous bursts that, importantly, are capable of perturbing ongoing ictal discharges (Shiri and others 2017). Thus, low-frequency stimulation of interneurons mediating either somatic or dendritic inhibition, by entraining synchronous discharges of large populations of interneurons, may impose longer refractory periods between bursts and thereby disrupt ictal activity. In contrast, high-frequency stimulation of PV+ neurons specifically leads to seizure prolongation (Wang and others 2017). Low-voltage high-frequency signals in the beta/gamma range are a landmark of the tonic phase of seizure (de Curtis and Avoli 2016), suggesting that high frequency synchronization may play an important role in seizure maintenance. It is perhaps not surprising, then, that high-frequency stimulation of fast spiking PV+ cells, which are known to play a key role in synchronizing pyramidal neuron activity and generating high-frequency gamma oscillations (Cardin and others 2009; Cobb and others 1995; Sohal and others 2009), might lead to prolonged seizure activity. Several interneuron-mediated mechanisms may be involved in maintaining this high synchronicity, such as synchronous inhibitory post-synaptic potentials on pyramidal neurons (akin to a mechanism supporting gamma oscillations—Principal INterneuron Gamma; PING), or synchronous interactions between interneuronal networks (INterneuron Gamma; ING) (for review, see Jiruska and others 2017). Whether high-frequency PV+ cell activation synchronizes the epileptic network during the tonic phase of seizures, however, remains to be established.

##### Location/timing of stimulation and seizure propagation

As previously discussed (see Seizure Initiation), the GABAergic system may be compromised in epileptic networks, leaving excitation unchecked and free to invade neighboring areas. This “inhibitory restraint” hypothesis (Box 2) raises the interesting possibility that GABAergic cells within the seizure focus, and those outside of the focus but in its penumbra, may be in distinct pathophysiological states, and thus respond differently to optogenetic manipulation. To test this directly, Sessolo and others (2015) puffed NMDA onto cortical slices perfused with 4-AP in order to create an identifiable micro-focus and assessed the effect of photo-stimulating PV+ interneurons inside or outside of this focus. Strikingly, they found that whilst PV+ cell photo-stimulation within the focus prolonged seizure duration, stimulation of these cells outside of the focus reduced it. Thus, the location of optogenetic intervention relative to the focus appears to play a key role in determining whether interneuron stimulation promotes or prevents seizure maintenance (Fig. 4B).

Given that seizure propagation is time-dependent, we can infer that the timing of stimulation, relative to seizure onset, will also be an important factor (Fig. 4C). Namely, when stimulating at the focus, photo-depolarization of interneurons could in theory still be anti-ictogenic if stimulation occurs immediately after seizure onset, before the focus has expanded, and the number of compromised GABAergic cells has increased; in this case, the area of illumination may still contain sufficient unaffected interneurons, able to respond to light stimulation and thus constrain the seizure. Indeed, several studies appear to support this hypothesis. For example, PV+ neuron photo-stimulation at the focus just after seizure onset was found to reduce seizure duration in an acute in vivo model using topical 4-AP application (Assaf and Schiller, 2016). Similar results were also seen in a chronic in vivo kainate model of TLE (Krook-Magnuson and others 2013). In contrast, two studies using generalized seizure models reported a pro-epileptic role of PV+ cells in seizure maintenance regardless of the timing of the optogenetic manipulation (Khoshkhoo and others 2016; Wang and others 2017). However, we hypothesize that during generalized seizures the cortical network features a compromised GABAergic system resembling that seen at the focus in focal seizure models, and thus attempts to further activate interneurons by photo-stimulation has similar pro- rather than anti-epileptic effects. Indeed, a recent study on pilocarpine-induced focal seizures revealed a rapid switch from an anti- to a pro-epileptic effect of PV+ interneuron photo-stimulation: photo-depolarization reduced seizure duration when triggered at the onset of a seizure but prolonged it when delayed by a few seconds (Magloire and others 2018). This suggests that the role of interneurons in seizure maintenance is dynamic and evolves as seizures progress in space and time.

##### Involvement of different GABAergic cell subtypes

Finally, the identity of the targeted GABAergic cell subtype is an important factor in determining whether optogenetic manipulation during seizures will promote or prevent seizure maintenance. Indeed, while PV+ cell activation appears to exert either anti- or pro-ictal effects, depending on stimulation frequency and location (see previous sections), stimulation of SOM+ interneurons seems to be, for the most part, inhibitory. Thus, both low- and high-frequency photoactivation of these cells was found to reduce seizure duration (Shiri and others 2017; Wang and others 2017). Furthermore, photo-inhibition of VIP+ interneurons, which almost exclusively inhibit SOM+ cells (Pfeffer and others 2013) and would therefore be expected to increase SOM+ cell activity, was also found to curtail seizures (Khoshkhoo and others 2016). Surprisingly, in the same study, Khoshkhoo and colleagues also found that photo-inhibition of neocortical SOM+ interneurons could reduce seizure duration, suggesting that these interneurons can also actively contribute to ictogenesis. Importantly, however, SOM+ interneurons represent a heterogeneous population, with some subtypes mediating disinhibitory instead of inhibitory effects (see Boxes 1 and 3), which may explain this discrepancy. It is also important to note that whilst photo-depolarization and photo-hyperpolarization constitute opposite optogenetic manipulations, they may not necessarily lead to opposite results. Indeed, photo-depolarization of SOM+ interneurons would be expected to have a net inhibitory action resulting from the simultaneous recruitment of many cells. In contrast, their photo-hyperpolarization will merely suppress their tonic activity, and the resulting network effect will therefore depend on the intensity of this activity and whether it is inhibitory or disinhibitory.

Box 3. Genetic markers group functionally distinct interneuronal populations.

While the development of interneuron-specific mouse Cre driver lines (Taniguchi and others 2011) has unquestionably greatly advanced our understanding of the role of interneurons within cortical circuits, it is important to note that the genetic markers used to target these cells often group different subclasses of interneurons (see Box 1), which may perform different, sometimes even opposing, functions.

PV+ interneurons, for instance, comprise not only fast-spiking BCs but also PV+ AA cells that unusually have been shown to elicit depolarizing post-synaptic responses, and even to evoke action potentials, in pyramidal neurons (Szabadics and others 2006; Woodruff and others 2009). This is hypothesized to be due to their unique innervation of the axon initial segment, where intracellular Cl^−^ concentrations are higher than at the pyramidal cell soma (Szabadics and others 2006). These paradoxical excitatory effects of PV+ AA cells have, however, not been found uniformly (Glickfeld and others 2009; Wang and others 2014), which may reflect methodological differences, and/or a dual, excitatory-inhibitory, cortical state-dependent role of these cells (Woodruff and others 2011).

SOM+ and VIP+ interneurons can also be divided into functionally distinct subsets. Indeed, while most SOM+ neurons, principally MCs, are involved in dendritic inhibition of pyramidal cells, a fraction of nMC SOM+ interneurons inhibit PV+ interneurons and thus perform a disinhibitory role (Xu and others 2013). Similarly, VIP+ cells, which are primarily involved in disinhibition, also comprise a subset of CCK+ BCs involved in perisomatic inhibition of pyramidal neurons (Tremblay and others 2016).

While these functionally divergent subtypes usually only make up a small proportion of the overall targeted group, they nevertheless need to be taken into consideration when interpreting results obtained using Cre-Lox technology. Additionally, it is important to note that some of these genetic markers are transiently expressed by principal cells early in development (e.g., CCK, Taniguchi and others 2011), limiting their use as interneuron-specific targeting tools.

While more work is evidently needed to fully understand the role of the various GABAergic cell subtypes in seizure maintenance, some clear differences are becoming apparent, in particular between the primarily anti-epileptic role of SOM+ cells and the more nuanced role of PV+ cells. As described above, the latter exert complex effects on seizure maintenance and propagation, which appear to evolve throughout the course of seizures (Fig. 4D). What mechanisms, specific to PV+ interneurons, might underlie these effects? Much evidence points toward PV+ interneurons as the primary cell-type involved in inhibitory restraint (see Box 2), and consequently also in its failure, leading to seizure onset and propagation. Thus, in rodent cortical slices, inhibitory barrages recorded in pyramidal cells shortly before the onset of ictal discharges were shown to coincide specifically with PV+, but not SOM+ cell burst spiking, and increases in PV+ cell activity during seizures were seen as far as 700 µm away from the seizure focus (Cammarota and others 2013), perhaps corresponding to the powerful inhibition described in the “ictal penumbra” (Schevon and others 2012; Smith and others 2016). During periods of intense activity, however, PV+ cells have also been found to enter into a state of depolarization block, thereby leaving runaway excitation unopposed (Cammarota and others 2013; Karlócai and others 2014). Indeed, Cammarota and others (2013) showed that fast-spiking PV+ interneurons, but not low-threshold spiking SOM+ interneurons, enter into depolarization block just before seizure onset. Such a failure of PV+ neuronal firing during seizures does, however, remain to be confirmed in vivo.

Another mechanism which may explain why PV+ cell activation becomes pro-epileptic as seizures progress is their participation in Cl^−^ loading of principal neurons (Fig. 3B). Indeed, by puffing GABA either at the soma or dendrites of pyramidal cells, Ellender and others (2014) found that a shift in E_GABA_ occurs predominantly in the perisomatic region, implicating PV+ BCs, which target this cellular compartment; furthermore, they showed that brief PV+ interneuron photo-activation at the end of epileptic after-discharges evoked depolarizing post-synaptic potentials. In line with these in vitro observations, we have recently demonstrated that overexpression of the KCC2 transporter in pyramidal cells, presumably reducing Cl^−^ loading, prevents the paradoxical seizure-promoting effect of delayed PV+ interneuron photo-depolarization in vivo (Magloire and others 2018). Interestingly, in addition to BCs, PV+ interneurons also include AA cells, which have been shown to generate depolarizing GABA_A_-mediated post-synaptic potentials in physiological conditions, explained by the absence of KCC2 at the axon initial segment of principal neurons (Szabadics and others 2006). AA cells may therefore also contribute to Cl^−^ loading, and hence to the pro-epileptic effects of PV+ neuron photo-stimulation. Thus, depolarization block and Cl^−^ loading are two potential mechanisms underlying the failure of somatic inhibition during seizure propagation which, importantly, are not mutually exclusive, and could work in tandem as seizures evolve.

## Conclusions and Future Challenges

In this review, we have outlined the possible roles of different GABAergic neuron subtypes in epileptiform activity, as identified by a growing body of work employing optogenetic and imaging tools. It is becoming apparent that their role is complex, and often varies through the different phases of epileptic events, from interictal activity and its transition to ictal discharges, to seizure maintenance and propagation. In spite of this, a few general conclusions can be drawn from this work. First of all, interneurons can trigger interictal and ictal activity, as demonstrated either by broad interneuron photo-stimulation or by specific activation of PV+ or SOM+ interneurons. Second, synchronization of interneurons plays a key role in the maintenance of epileptic activity; indeed, synchronous optogenetic activation of interneuron populations can either curtail or prolong seizures depending on the stimulation frequency used. Third, the ability of interneurons to suppress ictal activity depends on their location relative to the seizure focus, with their inhibitory power becoming compromised as the seizure spreads. Finally, the contribution of interneurons to restraining and/or promoting epileptiform activity is cell type-specific: recruitment of SOM+ cells, mediating dendritic inhibition, appears to generate broadly anti-epileptic effects, while the actions of activating perisomatic-targeting PV+ interneurons evolve from anti- to pro-epileptic effects as seizures progress.

A number of important questions, however, remain to be addressed. We still do not know, for instance, whether interneurons are necessary for the generation of interictal events in vivo, in the absence of acute experimental manipulations to disinhibit the circuitry. More important, despite the introduction of optogenetic tools, it is still unclear whether interneuron-mediated interictal activity promotes ictogenesis, or instead serves a protective function (Avoli and others 2002). To answer this, systematic photo-*inhibition*, rather than activation, of interneurons during interictal and ictal activity will be necessary to conclusively determine their role in seizures. Further investigation into interneuron synchronization and its involvement in seizure generation and maintenance is also warranted; de-synchronizing interneuron populations during seizures, for instance, by blocking electrical coupling, or by patterned optogenetic activation (e.g., holographic two-photon optogenetic stimulation; Yang and Yuste 2018), could provide significant insights. Finally, it is important to note that the majority of studies have thus far only focused on PV+, SOM+, and VIP+ interneurons. Other subtypes, such as CCK+ BCs and neurogliaform (NGF) neurons may also be involved in epileptiform activity, and thus hold promise as targets capable of preventing or curtailing seizures. Optimizing Cre driver lines specific for these subtypes will however be necessary to study their individual contributions to seizure activity.

A number of technical challenges need to be overcome in order to further advance our understanding of interneurons and their involvement in epilepsy, and how best to translate these findings into effective therapeutic strategies. For instance, identifying whether and when specific interneuron classes enter into dysfunctional states such as depolarization block will require the development of methods that allow us to follow their spiking behavior during seizures. Two-photon targeted juxtacellular and/or intracellular recordings of tagged interneurons are an option, although this has never been achieved during seizures in awake animals. If successfully applied, however, such methods could produce vital insights into the role of GABAergic cell subtypes in seizure initiation and maintenance. Another option is to use multi-electrode probes, together with spike sorting analysis, to record and identify individual neurons during seizures. This approach has the advantage of being transposable to chronic models, as well as to patients, although it does not yet allow one to conclusively identify recorded cells (Merricks and others 2015). A new high-density probe featuring 1000 contacts (Neuropixels), however, drastically increases the reliability of spike sorting analysis (Jun and others 2017), and, if combined with selective optogenetic activation of interneuron subtypes, may potentially allow to follow the spiking activity of single “optogenetically identified” neurons during seizures in spontaneous chronic seizure models.

Another important technical challenge lies in the identification and development of appropriate seizure models. Acute focal in vivo models have the advantage of creating stereotypical seizures on demand, with well-defined foci, making them suitable to investigate seizure mechanisms. In contrast, chronic models with spontaneous seizures represent the most translatable option, both to understand the role of GABAergic cells in human epilepsy and to test potential therapeutic approaches. However, seizures are generally unpredictable and less frequent in these models, and their foci are often difficult to identify. Fortunately, new chronic models with focal seizures are beginning to emerge, such as intracortical injection of tetanus toxin (Pitkänen and others 2005) or induction of focal cortical dysplasia by in utero electroporation of genes, somatic mutations of which underlie this disorder (Hsieh and others 2016).

Resolving these technical challenges, and extending the investigation to other GABAergic cell subtypes, will greatly advance our understanding of the role of interneurons in epilepsy, and how best to harness them to curtail and prevent seizures.
